# Predicting short-term interruptions of antiretroviral therapy from summary adherence data: Development and test of a probability model

**DOI:** 10.1371/journal.pone.0194713

**Published:** 2018-03-22

**Authors:** Rebecca Arden Harris, Jessica E. Haberer, Nicholas Musinguzi, Kyong-Mi Chang, Clyde B. Schechter, Chyke A. Doubeni, Robert Gross

**Affiliations:** 1 University of Pennsylvania School of Medicine, Philadelphia, Pennsylvania, United States of America; 2 Harvard Medical School, Boston, Massachusetts, United States of America; 3 Massachusetts General Hospital, Boston, Massachusetts, United States of America; 4 Mbarara University of Science and Technology, Mbarara, Uganda; 5 Philadelphia VA Medical Center, Philadelphia, Pennsylvania, United States of America; 6 Albert Einstein College of Medicine, Bronx, New York, United States of America; Azienda Ospedaliera Universitaria di Perugia, ITALY

## Abstract

Antiretroviral therapy (ART) for HIV is vulnerable to unplanned treatment interruptions–consecutively missed doses over a series of days–which can result in virologic rebound. Yet clinicians lack a simple, valid method for estimating the risk of interruptions. If the likelihood of ART interruption could be derived from a convenient-to-gather summary measure of medication adherence, it might be a valuable tool for both clinical decision-making and research. We constructed an *a priori* probability model of ART interruption based on average adherence and tested its predictions using data collected on 185 HIV-infected, treatment-naïve individuals over the first 90 days of ART in a prospective cohort study in Mbarara, Uganda. The outcome of interest was the presence or absence of a treatment gap, defined as >72 hours without a dose. Using the pre-determined value of 0.50 probability as the cut point for predicting an interruption, the classification accuracy of the model was 73% (95% CI = 66%– 79%), the specificity was 87% (95% CI = 79%– 93%), and the sensitivity was 59% (95% CI = 48%– 69%). Overall model performance was satisfactory, with an area under the receiver operator characteristic curve (AUROC) of 0.85 (95% CI = 0.80–0.91) and Brier score of 0.20. The study serves as proof-of-concept that the probability model can accurately differentiate patients on the continuum of risk for short-term ART interruptions using a summary measure of adherence. The model may also aid in the design of targeted interventions.

## Introduction

With more potent and safe antiretroviral (ARV) therapies increasingly available, HIV-infected individuals are living longer than ever before [1]. For many patients, however, it is difficult to maintain the day-to-day consistency in adherence necessary to achieve viral suppression over the life-time course of the disease [2]. Treatment interruptions–consecutively missed doses over a series of days–are a common occurrence [3]. Research has found that interruptions increase the risk of viral replication [4], drug resistance [5], and disease progression [6]. Interruptions also create a window for the spread of new infections [7] and may prolong the endemic stability of HIV in many communities [8].

Clincians lack a simple, valid method for estimating the current risk of interruptions. Average adherence is a commonly used summary measure of ARV regimen adherence that can be objectively determined from pharmacy refill or pill count data [9]; however, as a indicator of whether drug concentrations are sufficient to maintain viral suppression, average adherence can be insensitive to the pattern of missed doses [10]. For example, an average adherence of 0.90 could reflect 1 missed dose every 10 days, or it could reflect a 6-day run of missed doses over 60 days. Therapeutic drug levels are likely to be less affected by scattered lapses in adherence than by a sustained interruption, which in turn increase risk of virologic breakthrough [11]. At present, electronic adherence monitoring (EAM) devices–pill containers that electronically record the date and time of each opening–are the instruments most often used to quantify the precise duration of treatment interruptions [9]. Too resource-intensive for routine clinical care, the devices are used when highly detailed data are needed to answer a research question. But if the likelihood of antiretroviral therapy (ART) interruptions could be accurately estimated from a summary measure using readily available data [12], interventions to prevent treatment interruptions could be tailored to the level of individual risk. Therefore, we aimed to construct a probability model of ART interruptions based on an individual’s average adherence and to assess the predictive performance of the model.

To maximize clinical relevance, we focused on short-term interruptions, defined as zero adherence for 72 hours continuously. Short strings of missed doses are more common than lengthy interruptions [10], and their association with virologic breakthrough has been firmly established [4,5,13–16].

## Methods

### Construction of the probability model

In the probability theory literature on discrete random variables, the sample space for many games of chance is the set of 2 mutually exclusive outcomes: success or failure. A string of consecutive failures is represented in standard terminology as a failure run of length *r* in *n* trials [17]. Applying the terminology to ARV treatment interruptions, we define an event of “adherence failure” as any 24-hour period (1 day) in which no ARV dose is taken. A run of consecutive days without an ARV dose is termed a treatment interruption of length *r*. Our objective is to determine the probability, by level of average adherence, of at least one treatment interruption of length *r* in an observation period of *n* days. In the absence a closed-form equation, we use Feller’s approximating formula from general run theory [18]. The probability of at least one treatment interruption is given by

$$Prob(TI)\approx 1-\left[\frac{1-px}{(r+1-rx)q}∙\frac{1}{x^{n+1}}\right]$$ (1)

where:

*TI* = at least one treatment interruption,
*r* = length of treatment interruption in days,
*n* = number of days in the observation period,
*p* = probability of adherence failure on any given day,
*q* = probability of adherence success on any given day, (*q* = 1 –*p*). The unbiased estimate of *q* is average adherence, and
*x* = smallest positive root of 0 = 1 –*x* + *qp*^*r*^ *x*^*r* + 1^

For any sufficiently large sample of patients [19], the model predicts the proportion of patients who will experience an ARV treatment interruption of length *r* in a known interval of time *n* by level of average adherence *q*. (See S1 Appendix for supplemental information on Eq (1) and S2 Appendix for the sample size requirements of prediction models.)

We theorize the model will yield the most accurate predictions under the following conditions:

1. *Non-prescribed interruptions*. An unstructured treatment interruption is a patient-initiated temporary discontinuation of all ART drugs after which treatment is resumed. It excludes planned treatment interruptions overseen by a physician for medical reasons such as drug toxicity, suppression failure, or drug-resistance [3].
2. *Unencumbered access to medications*. The model is not applicable to situations where a patient does not have continuous access to ARVs. Lack of access might occur for reasons of pharmacy stock shortages, individual resource constraints, or other circumstances [20,21].

### Study setting and participants

We tested the model with data collected on185 HIV-infected individuals initiating ARV therapy in a prospective observational cohort study in southwestern Uganda beginning in 2005 and ending in 2011. The Uganda AIDS Rural Treatment Outcomes (UARTO) study recruited participants from a public clinic, the Immune Suppression Syndrome Clinic at the Mbarara Regional Referral Hospital, which dispenses free ARV therapy in the region. Patients greater than 18 years old and residing within 60 km from the clinic were eligible for study participation [22].

The data were collected with ethical approval from Mbarara University of Science and Technology, Uganda National Council for Science and Technology, Partners Healthcare/Massachusetts General Hospital, and University of San Francisco California. All participants gave written informed consent.

### Adherence and interruption measures

ARV adherence was measured using MEMS (WestRock, Switzerland), an EAM in a bottle cap. Participant’s EAM data were downloaded monthly at home visits that were for data collection only. Average adherence was computed as the number of EAM openings divided by the prescribed number of doses in the first 90 days of therapy. Treatment interruption was operationally defined as zero adherence for >72 hours continuously at any point in the first 90 days of therapy.

To preclude results that would exaggerate the performance of our model, we excluded observations of average adherence <0.333 (n = 113) and ≥0.967 (n = 7) because, respectively, these participants either always experience or never experience 3-day interruptions in a 90-day timeframe. Thus, modeling the probability of interruption for these groups was unnecessary. We also excluded average adherence from 0.333 to 0.39 because the number of observations in this interval was sparse (n = 3). This left 185 observations for analysis with average adherence of participants ranging from 0.40 to 0.966. See S3 Appendix for the sensitivity analysis of excluded cases.

To analyze the frequency of interruptions by level of adherence, we grouped the 185 participants’ average adherence values into intervals. As the adherence distribution was asymmetrical with a negative skew (skewness = -1.04), we used wider intervals for the two lower levels of adherence (0.40 –<0.50, 0.50 –<0.60), narrower intervals in the mid- and upper-range of adherence (0.60 –<0.65, 0.65 –<0.70, 0.70 –<0.75, 0.75 –<0.80, 0.80 –<0.85, 0.85 –<0.90), and the narrowest intervals at the highest range where a larger number of observations were concentrated (0.90 –<0.934, 0.934 –<0.967). The grouping facilitated a more detailed examination of the adherence-interruption curve in the segments where most of the data points were concentrated.

### Socio-demographic and health functioning measures

At the enrollment visit, socio-demographic and economic information was collected from each participant. Baseline data were also collected on potential confounders, including self-reported distance from clinic (in minutes of travel-time) [23], screen for heavy drinking (3-item consumption subset of the Alcohol Use Disorders Identification Test) [24], depression symptom severity (15-item Hopkins Symptom Checklist for Depression, modified for the local context with the addition of a 16th item, “feeling like I don't care about my health”) [25], and CD4.

### Statistical analysis

We used visual inspection and graphical analysis to determine the shape of the empirical curve relating average adherence to ART interruptions. To determine whether the interruptions observed at each interval of adherence differed from the hypothesized values of the probability model, we computed exact p-values using the binomial test for goodness of fit [26]. A finding of nonsignificance indicated a satisfactory fit to the data.

Several criteria were used to evaluate the overall classification and prediction performance of the probability model. We *a priori* specified the inflection point of the prediction curve–the point on the curve where the probability of an ART interruption equals 0.50 –as the cut point to calculate the sensitivity, specificity, and percent correctly classified. Less than 0.50 predicted no treatment interruptions of 3 days or more, ≥0.50 predicted at least one treatment interruption. The 95% confidence limits for the classification statistics are Clopper-Pearson intervals [26]. To measure the total difference between the model predictions and observed outcomes, we used the Brier score which can range from 0 for a perfect model to ≥0.25 for a non-informative model [27]. We evaluated the model’s discriminative ability using the area under the receiver operating characteristic curve (AUROC). AUROC values range from 0.5 to 1.0, with values ≥ 0.7, ≥ 0.8, and ≥ 0.9 considered as satisfactory, good, and excellent, respectively [28]. To gauge the robustness of the AUROC estimate, we used *k*-fold cross-validation, with *k* = 10. The data set was randomly split into 10 roughly equal-sized parts. A candidate model was developed based on 9 parts of the data set. The AUROC of the candidate model was then evaluated on a test set containing the data in the hold-out part. Using each of the 10 parts as the test set and repeating the model building and evaluation procedure, the results from the folds were then averaged to obtain the cross-validated AUROC estimate [29].

We fitted regression models with the baseline demographic and clinical variables to identify potential confounders or a secular trend affecting the frequency of interruptions [30]. Confounding effects were judged to be present if the point estimate of the association between the probability model prediction variable and ART interruptions changed by >15%. Outlying data points were identified and included in all analyses.

All tests of significance were two-sided, with p <0.05 used as the threshold of statistical significance. The statistical analysis was performed using Stata version 15.0 (StataCorp, College Station, TX).

## Results

### Hypotheses generated by the model and tested using UARTO data

From the model, we derived the following hypotheses:

1. *The probability of an interruption is a S-shaped decreasing function of average adherence*.
  More specifically, the model predicts a sigmoid curve where the rate of change in the probability of interruptions with respect to average adherence is characterized by a bell-shaped distribution, approximating 0 at both tails of adherence, and reaching a maximal plateau in the region surrounding the inflection point. The inflection point corresponds to the 0.50 predicted probability of interruptions which, for *r* = 3, *n* = 90, occurs at 0.79 adherence.
2. *The expected number of persons with at least one ART interruption of three days or more by average adherence interval is presented in Table 1*.

**Table 1:** Expected number of persons with at least one ART interruption of three days or more by average adherence.

Average Adherence	Expected Frequency of Interruptions per 100 persons	Number of Participants in UARTO Cohort	Expected Frequency of Interruptions in UARTO Cohort
0.934–0.967	1	39	0
0.90 –<0.934	5	37	2
0.85 –<0.90	14	25	4
0.80 –<0.85	33	16	5
0.75 –<0.80	55	19	10
0.70 –<0.75	76	11	8
0.65 –<0.70	89	10	9
0.60 –<0.65	96	8	8
0.50 –<0.60	100	12	12
0.40 –<0.50	100	8	8

The mid-point of the adherence intervals provided the *q* parameter input for the probability model equation (*q*, *r* = 3, *n* = 90).

### Participant characteristics

The 185 participants were observed for a period of 90 days each. At enrollment, 123 (66%) of participants were female, 75 (41%) were married, 148 (80%) were literate, and 138 (75%) had some form of employment. Median age was 34 years (IQR = 28–39). The most common ART regimens at initiation were Zidovidine/Lamivudine/Nevirapine, Stavudine/Lamivudine/Nevirapine, and Zidovudine/Lamivudine/Efavirenz, consisting of two tablets per day, used by 114 (62%), 46 (25%), and 19 (10%) of participants, respectively. Median baseline CD4 T-lymphocyte count was 138 cells/mm^3^ (IQR = 83–201). The adherence average for all participants was 0.815 (SD = 14.5), the median was 0.872 (IQR = 0.738–0.923). Over the 90-day interval, 93 of the 185 participants (50.3%) had zero treatment interruptions ≥3 days and 92 (49.7%) had one or more (Table 2).

**Table 2:** Participant characteristics (N = 185).

Characteristics	*Freq*.	*%*
Female	123	66.5
Education		
None	25	13.5
Primary	116	62.7
Secondary	44	23.8
Literate	148	80.0
Unemployed	47	25.4
Married	75	40.5
Alcohol use disorder^a^	23	12.9
Depression	64	34.6
ARV regimen^b^		
Zidovidine/Lamivudine/Nevirapinee	114	62.3
Stavudine/Lamivudine/Nevirapine	46	25.1
Zidovudine/Lamivudine/Efavirenz	19	10.4
Other ARV regimens*	4	2.2
ART interruption ≥ 3 days	92	49.7
	*Mean / Median*	*IQR*
Age	34 / 34	28–39
CD4 cell count^c^	154 / 138	83–201
Travel time to clinic (minutes)	56 / 40	20–60
Average adherence	0.815 / 0.872	0.739–0.923

^a^Six missing values

^b^ two missing values

^c^eight missing values

*Stavudine/Lamivudine/Efavirenz, Zidovudine/Nevirapine/Tenofovir, Efavirenz/Emtricitabine/Tenofovir, Emtricitabine/Nevirapine/Tenofovir.

### Model performance: Comparing expected and observed interruptions

**Fig 1:**
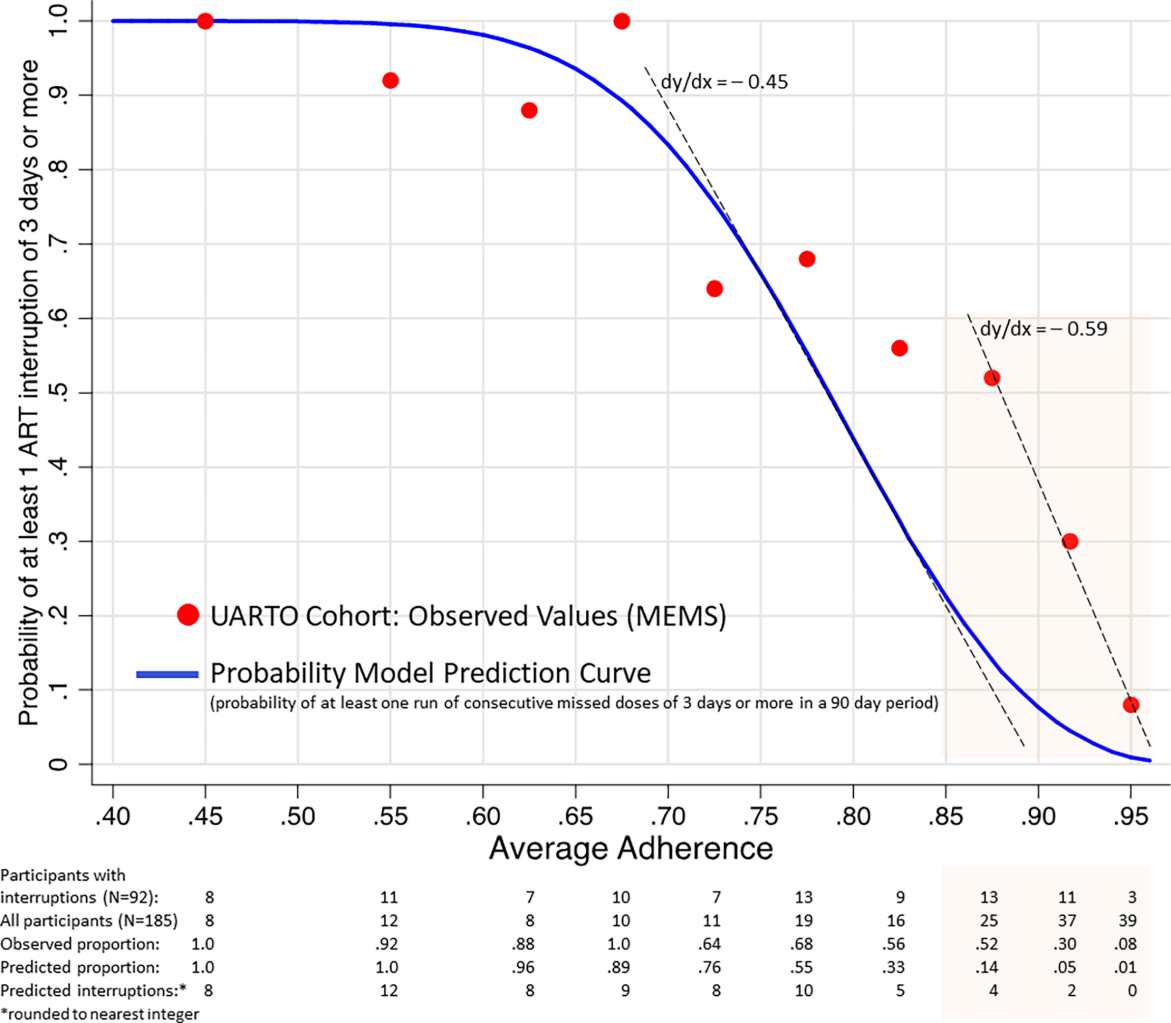
Comparison of observed and predicted values of ART interruption by average adherence.

Fig 1 displays the relationship between average adherence and the probability of at least one treatment interruption of ≥3 days. From 0.40 through 0.85 average adherence, the UARTO cohort data follow the sigmoid contours of the prediction curve. The expected slow decrease in the rate of change from 0.40 through 0.65 adherence is present, as is the sharp decline from 0.65 adherence through the inflection point to 0.80 adherence. Beyond this point, the probability model anticipates that the frequency of interruptions would continue to fall rapidly and then level off toward 0. However, the three data points in the upper adherence intervals (0.85 –<0.90, 0.90 –<0.934, 0.934 –<0.967) show higher interruption frequencies than expected (shaded region).

To assess the empirical fit of the model, we compared the cohort data with model predictions at each adherence interval. The results are presented in Table 3. At the mid- and lower levels of adherence, <0.85, the model performs generally well with none of the difference tests statistically significant (0.40 –<0.50, p = 0.99; 0.50 –<0.60, p = 0.11; 0.60 –<0.65, p = 0.28; 0.65 –<0.70, p = 0.61; 0.70 –<0.75, p = 0.31; 0.75 –<0.80, p = 0.26; 0.80 –<0.85, p = 0.06). At the higher levels of adherence, 0.85 –<0.967, the model underpredicts the proportion of participants who experience an interruption. Of the three outliers, the departure from expectation is greatest at 0.85 –<0.90 adherence where 13 of the 25 participants (0.52) had interruptions and 4 (0.14) were expected (p <0.01). The model predicted a falling off in the rate of interruptions, but the descent does not occur until 0.90 adherence at which point the decline, as measured by the linear slope of the three outliers (dy/dx = -0.59, p = 0.039), is more abrupt than the slope of the prediction curve (dy/dx = -0.45) (Fig 1).

**Table 3:** Comparison of the predicted and observed proportion of participants with at least one ≥3 day ART interruption in the course of 90 days (*r* = 3, *n* = 90) by average adherence.

Average Adherence (*q*)	Probability of at Least One Interruption of 3 Days or More in 90 Days		p-value*
	Predicted	Observed	
0.934 –<0.967	0.01	0.08	<0.01
0.90 –<0.934	0.05	0.30	<0.01
0.85 –<0.90	0.14	0.52	<0.01
0.80 –<0.85	0.33	0.56	0.06
0.75 –<0.80	0.55	0.68	0.26
0.70 –<0.75	0.76	0.64	0.31
0.65 –<0.70	0.89	1.00	0.61
0.60 –<0.65	0.96	0.88	0.28
0.50 –<0.60	1.00	0.92	0.11
0.40 –<0.50	1.00	1.00	0.99

* Two-sided p-values were computed using the exact binomial test for goodness of fit. The midpoint of the adherence intervals provided the *q* parameter input for the probability model generating the point predictions.

**Fig 2:**
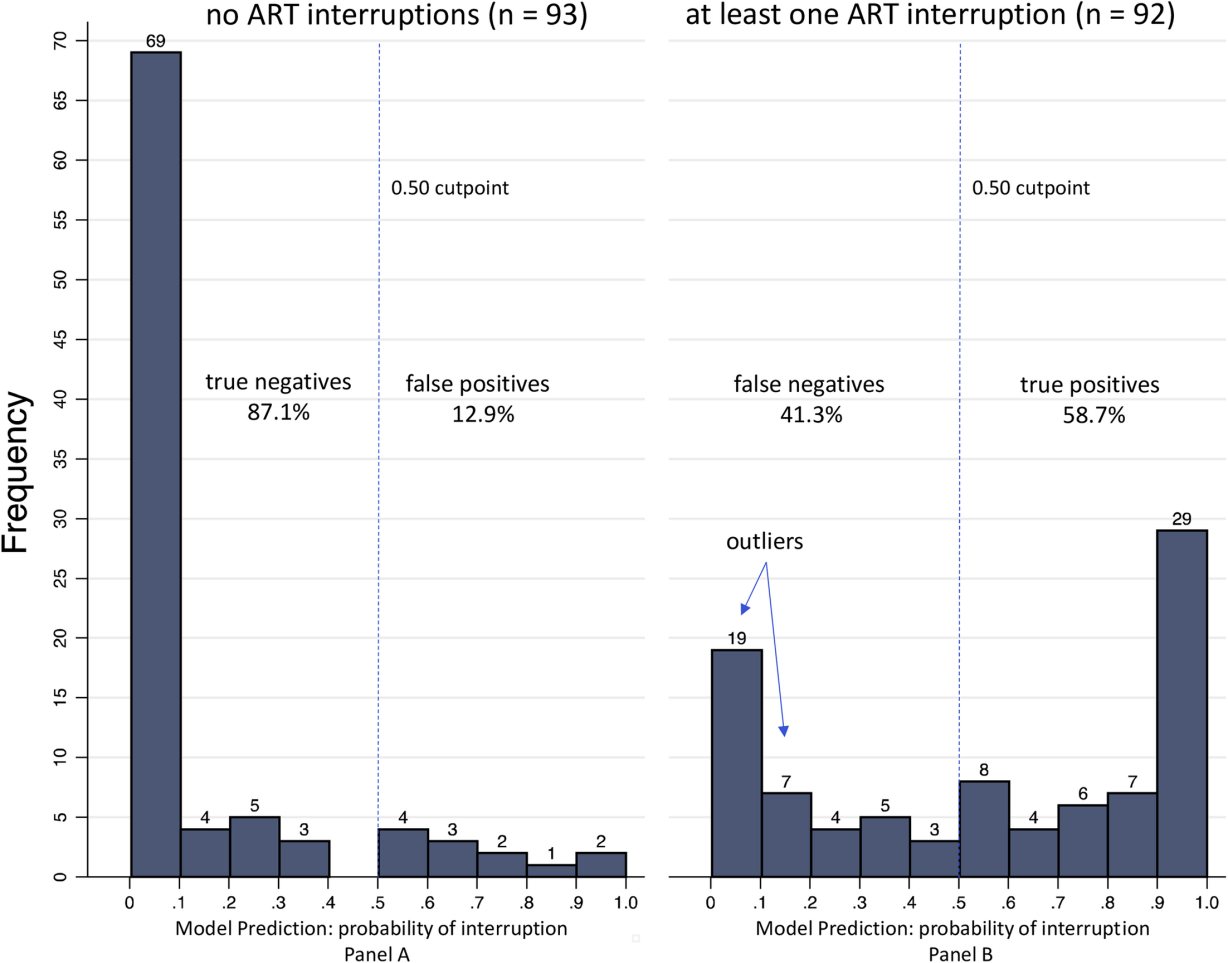
Distribution of model prediction variable by interruption status. Panel A: histogram of the prediction variable for subset of participants who did not have an ART interruption (n = 93). Panel B: histogram of the prediction variable for subset of participants who had at least one ≥ 3-day ART interruption (n = 92). Outliers refer to the larger than expected number of participants with a low probability of interruption (≤0.20) who experienced at least one interruption. Pre-determined classification cutpoint for both participant subsets was 0.50. Specificity [true negatives / (true negatives + false positives)] = 87.1%. Sensitivity [true positives / (true positives + false negatives)] = 58.7%. The classification accuracy of the probability model [(true negatives + true positives) / total N] was 73.0%. Outliers were included in the calculations.

**Fig 3:**
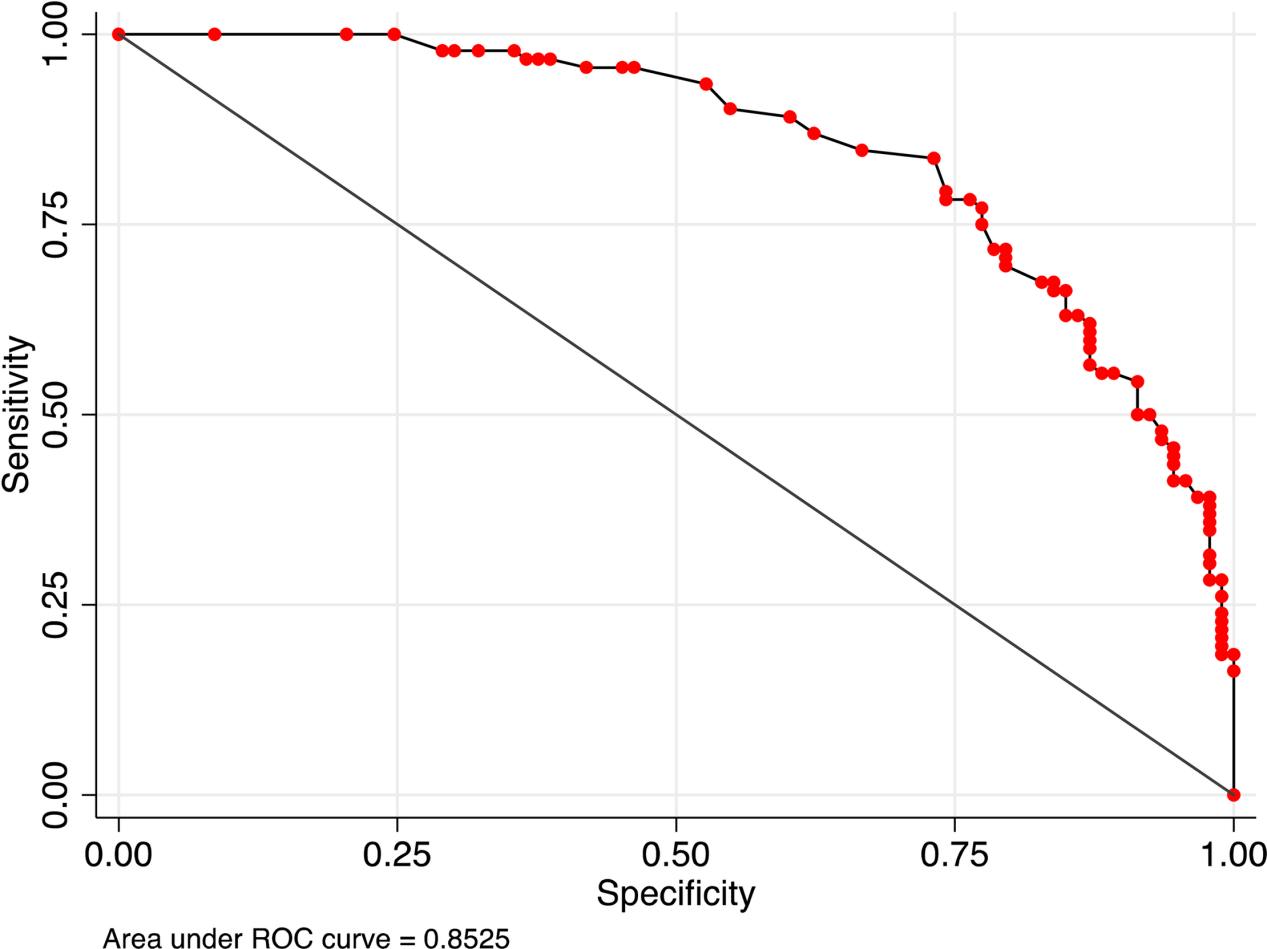
Receiver operating characteristics curve.

The histograms of the prediction variable for the participants who did (and did not) have an ART interruption ≥3 days are displayed in Fig 2. Using the pre-determined value of 0.50 as the cut point for identifying an interruption, the distributions show that the model has superior specificity (87%, 95% CI = 79%– 93%, panel A), but moderate sensitivity (59%, 95% CI = 48%– 69%, panel B) partly due to the outliers (false negatives) below 0.20 on the prediction scale. The classification accuracy of the probability model was 73% (95% CI = 66%– 79%). Allowing the cut point to vary, the model AUROC was 0.85 (95% CI = 0.80–0.91) and the *k*-fold cross-validation was 0.84 (95% CI = 0.78–0.90). The maximum classification accuracy for any cut point was 78% (95% CI = 71%– 83%), which was located at 0.89 average adherence. The receiver operating characteristic curve is displayed in Fig 3.

Examining overall performance, the Brier score for our prediction variable was 0.20. For comparison, we also examined the prediction performance of average adherence. The Brier score was 0.29, indicating that average adherence did not predict short-term interruptions.

No confounding effects (see S4 Appendix) or secular trends (S5 Appendix) were identified in the statistical analysis.

## Discussion

The probability model of treatment interruption based on average adherence demonstrated satisfactory performance in classification metrics, global fit, and discriminative ability. Short-term ART interruptions generally followed the prediction curve. At the mid-to-upper levels of adherence, we hypothesized and the data substantiated that small increases in average adherence would produce large decreases in the frequency of interruptions. This finding suggests the possibility of greater returns on efforts to improve adherence in the region surrounding the inflection point. Interestingly, the probability model predicted that the sharpest drop in the frequency of interruptions would occur at a lower level of adherence than found in the UARTO data. Also, the adherence–interruption slope was slightly more vertical in the UARTO data than expected. In all, we found that the probability model could distinguish individuals on a continuum of risk for short-term treatment interruptions. Sensitivity analysis and cross-validation confirmed the robustness of the results.

A probability model that provides a satisfactory fit to the data has traditionally been open to two interpretations. The first is that a model derived from a probability theorem represents a null hypothesis, and that observations which do not depart from expectation are said, therefore, to be consistent with chance. The second interpretation is that a balance of forces is present that mimics the chance process defined by the null hypothesis [31]. We have not found evidence of positive and negative effects in balance, and are reluctant to label short-term interruptions as simply “random.” We are modeling interruptions at the incipient stage, the period before the lengthier strings of nonadherent days have emerged. We conjecture that longer strings are associated with clinical and behavioral causes such as alcohol abuse or depression [32]; however, in this study of short-term interruptions, no such associations were found.

The definition of “short-term” varies in interruption research, although a common definition is a run of consecutively missed doses over 3 or 4 days. Definitions of “long-term” interruptions are more heterogeneous. At the low end, a run of 15 or 30 days of consecutively missed doses is commonly considered long-term; at the higher end, a run of several months [3]. We have focused our model on short-term interruptions as they are the occasion of most suppression failures. Consistent with the theory of runs [33], short-term interruptions occur more frequently than long-term interruptions, and the greater frequency is sufficient to offset the higher rate of viral rebound arising from long-term interruptions–patterns evident in the UARTO data [10]. Thus, models of short-term interruptions may be especially apt in resource-limited settings where second-line treatment regimens are often not available or affordable [34].

Our model fills an important gap, a means of predicting short-term interruptions using average adherence as an input parameter. We also note that our study found that average adherence, when regarded as an independent variable apart from the probability model equation, lacked the ability to predict interruptions. One reason is that the rate of change in the slope of the adherence–interruption relationship varies with the parameters *r* and *n*, the run length of missed doses and the period of observation, respectively. By itself, average adherence does not carry sufficient information to make exact predictions of ART interruptions.

As a prediction instrument, our model has several notable strengths. Because it is a straightforward application of probability theory (and not developed from any particular data set), the inductive two-sample development and external validation approach to instrument construction was unnecessary. When tested against observations, prediction accuracy was not subject to shrinkage requiring a “correction for optimism” [35]. Another strength of the model is the strategic information it provides to clinicians and planners for optimizing scarce case management resources. For example, while any non-adherence is concerning, the instrument can help center attention on those patients for whom modest improvements in average adherence are likely to produce large decreases in interruptions. As noted, the interval surrounding the inflection point is the optimal target since at this amount of adherence, the smallest increases (e.g., induced by an intervention) yield the greatest reduction of interruptions. In our study, patients who increase average adherence by 0.10, from 0.74 to 0.84, would reduce their interruption probability from 0.70 to 0.26. By contrast, at the lower levels of adherence, a comparable improvement in adherence will not produce a meaningful drop in interruptions–e.g., an increase in adherence from 0.60 to 0.70 will reduce the interruption probability from 0.98 to 0.83. For these patients, a qualitatively different type of intervention may be needed, one that might deploy a comprehensive network of supports. Familiarity with the adherence–interruption relationship might aid in the design of targeted interventions.

In the UARTO data, a higher average adherence value (0.89) was found for the binary classification cut point than was predicted by the model (0.79), suggesting that intervention resources might be allocated to patients at the upper levels of adherence as well. We caution that such an adjustment may be premature. For any cut point, the maximum classification accuracy was 78%, which was within the confidence interval observed for our pre-determined cut point (95% CI = 66%– 79%). The post-hoc selection of a cut point usually overstates diagnostic utility when applied to other samples [36–38]. A retest of the model using a different study population will help determine whether the departures from prediction require model calibration.

The identification of “high-yield” intervention points entails a tradeoff between sensitivity and specificity, between the “false calls” and “missed calls” of treatment interruption, an area of decision analysis presently unexplored in ART interruption studies. In quantifying the likelihood of a treatment gap, our model provides a framework for a more explicit examination of tradeoffs. We stress, however, that the instrument is not prescriptive. Clinicians should consider the circumstances and unique needs of each patient, including the likelihood of ART interruption, when deciding whether to mobilize adherence supports. No single adherence intervention or package of interventions will likely be effective for all populations and all settings.

For most patients, the consistent level of adherence needed to suppress viral replication over a lifetime remains a major challenge. Clinicians seldom know the extent that individuals are at risk of an interruption. Even people with excellent adherence who typically meet the optimal target of ≥0.95 average adherence, or ≤1 one missed dose per month, will occasionally experience treatment interruptions owing to inevitable disruptions in daily routine, onset or worsening of a co-morbid condition, or simple pill fatigue [39]. Lower levels of adherence (0.85–0.90) that elicit only minor decreases in viral load suppression have been demonstrated with improved, second-generation ARV formulations, e.g., non-nucleoside reverse transcriptase inhibitors [40], but generalizability remains undefined [41] and may depend on prior suppression time [11]. For the time being, perfect adherence continues to be the goal for every patient.

Our model provides a new understanding of the relationship between average adherence and the risk of short-term ART interruptions, which we tested for *r* = 3, *n* = 90. For each value of average adherence there is a corresponding interruption probability. The relationship can be displayed as a table or graph as in Fig 1. In settings where objective adherence measurement is a component of clinical practice, the instrument is a ready fit. However, some front-line facilities may not measure adherence [42], or measure precisely, and operations in those settings may need to be augmented. We also expect that each health facility will undergo a brief “learning curve” to find the right balance between risk and resource expenditures. Using the prediction instrument, clinicians and team members will see at a glance where each patient falls on the risk curve, and depending on patient circumstances and needs, and the adherence supports available (e.g., individual or group counseling, adherence training, mobile phone reminders, case managers, peer support), will find opportunities to increase cost-effectiveness by aligning services to risk.

A limitation of this study is the possibility of an observer effect [43,44]. EAM may alter the medication-taking behavior of some participants, the devices providing a visual reminder that adherence patterns are being observed, although the evidence to date suggests that if present, the effect is small [45]. Data collected through EAMs are also subject to measurement error since the caps can be opened and closed without taking any medication (“curiosity checks,” influence of social desirability) and more than one dose can be removed when a bottle is opened (“pocket dosing”) [9,46].

In conclusion, our study serves as proof-of-concept that the probability model can accurately differentiate patients on the continuum of risk for short-term treatment interruptions. It may also aid in the design of targeted interventions. However, to be a practical tool for clinicians, the model will need to be validated using more accessible data sources for measuring average adherence than EAM. An assessment of the predictive performance of the model using pharmacy records would be an appropriate next step. Average adherence based on refill data is a valid, simple to calculate measure easily incorporated into clinical practice [12, 47]. The adherence calculations can be automated in tandem with our prediction instrument. This strategy is ripe for testing.

## Supporting information

S1 Appendix Background information on Eq (1). (DOCX)

S2 Appendix Sample size requirements. (DOCX)

S3 Appendix Excluded cases and sensitivity analysis. (DOCX)

S4 Appendix Tests for potential confounders. (DOCX)

S5 Appendix Tests for secular trend. (DOCX)

## Abbreviations

ART: antiretroviral therapy
ARV: antiretroviral
AUROC: area under the receiver operating characteristic curve
EAM: electronic adherence monitoring
HIV: human immunodeficiency virus
MEMS: Medication Events Monitoring System
UARTO: Uganda AIDS Rural Treatment Outcomes
