# Supplementary material for: Predicting short-term interruptions of antiretroviral therapy from summary adherence data: Development and test of a probability model
Source: PLoS One. 2018 Mar 22;13(3):e0194713. doi: 10.1371/journal.pone.0194713 (PMC5864044; doi:10.1371/journal.pone.0194713)
Supplement: S1 Appendix — (DOCX) [file pone.0194713.s001.docx]

**S1 Appendix. Background information on Equation (1)**

Eq (1) is an application of Feller’s solution to a classical problem in probability [1]. Briefly stated, in the sample space with a set of 2 mutually exclusive outcomes, success *p* and failure *q*, what is the probability of a consecutive run of at least *r* successes in *n* trials? The problem is surprisingly complicated and intractable in terms of an exact solution. Feller’s equation is an approximation, but accurate to 2 decimal places for *n* as small as 4 and improves rapidly with increasing *n.* To our knowledge, this is the first application of the equation to ARV treatment interruptions.

We used Eq (1) to predict the relationship between average adherence and the probability of ARV interruptions of 3 days or more occurring over a span of 90 days. We chose the parameter values of *r* = 3 and *n* = 90 with particular virologic and clinical patterns in mind (viral replication resulting from brief adherence gaps; standard 3-month patient follow-up appointments). Depending on the question at hand, researchers might select other values of *r* and *n*, which will yield sigmoid prediction curves with different event probabilities. For example, if interest centers on longer failure runs, Eq (1) will predict fewer occurrences overall (analogy: chances of getting 7 *versus* 3 consecutive tails in 90 coin tosses) and the inflection point will be nearer to 0 adherence (i.e., complete non-adherence). If longer periods of observation are the focus, more failure runs will be predicted (chances of getting 3 consecutive tails in 120 *versus* 90 coin tosses) and the inflection point will be nearer to 1.0 adherence (i.e., perfect adherence). One cannot infer from the UARTO findings whether predictions will be close to actual outcomes when researchers employ different values of *r* and *n*.

The *x* term in Eq (1) warrants comment as it appears unrelated to the familiar *q*, *r*, *n* parameters. One of the intermediate steps in the construction of the formula is the derivation of a generating function of recurrence times, the denominator of which is the root of a polynomial. The *x* term is the unique positive root that satisfies the polynomial expression. For the details of this part of the mathematical argument, see Feller [1, pp 322-326].

1. Feller, W. An Introduction to Probability Theory and Its Applications. Vol 1. John Wiley and Sons, 1968.
