# Supplementary material for: Predicting short-term interruptions of antiretroviral therapy from summary adherence data: Development and test of a probability model
Source: PLoS One. 2018 Mar 22;13(3):e0194713. doi: 10.1371/journal.pone.0194713 (PMC5864044; doi:10.1371/journal.pone.0194713)
Supplement: S2 Appendix — (DOCX) [file pone.0194713.s002.docx]

**S2 Appendix. Sample size requirements**

For prediction models with binary outcomes, power is determined by the number of events, that is, by the effective sample size [1]. In the model development data set, too few outcome events relative to the number of candidate predictors are likely to yield unstable prediction models that are overfit to the sample and perform poorly on new data [2]. A widely-advocated rule of thumb for multivariable prediction models is a 10:1 ratio of the number of individuals with the outcome event to the number of candidate predictors, termed events per variable (EPV) [2]. Other guidelines have suggested an EPV as low as 5 or as high as 20 [3]. Investigating external validation samples, Vergouwe and colleagues found the EPV criterion inadequate, proposing instead a goal of 100 events and 100 non-events. Based on simulation studies, they found that substantially fewer cases could obscure small but potentially important differences in the estimation of performance measures [1]. Collins et al. concur [4]. A related consideration is the heterogeneity of case mix between the development and validation samples. With low case-mix heterogeneity, mainly statistical reproducibility can be inferred from predictor-outcome associations. Generalizability to plausibly related but untested settings (clinical transportability) requires dissimilar samples for development and validation [5].

Our prediction model differs from the models contemplated by these recommendations in two respects. First, our model is not multivariable. With only one predictor – the probability of a treatment interruption – our model is simpler. Consequently, the EPV of the UARTO data set was very high (EPV = 92). Of the 185 participants, 92 had at least one ART interruption ≥3 days and 93 participants had zero interruptions. Second, our prediction model was not developed from a training set, it was constructed *a priori* from a probability theorem. The concern with overfitting the development sample is not entirely applicable. Nor is the concern with the degree of relatedness in the case mix between the model development and validation samples. Still, our effective sample size came close to meeting the Vergouwe-Collins targets for external validation studies of multivariable models. In sum, the UARTO data with 92 events and 93 non-events provided an adequate first test of both the validity and transportability of our prediction model of ARV treatment interruption.

1. Vergouwe Y, Steyerberg EW, Eijkemans MJC, Habbema JDF. Substantial effective sample sizes were required for external validation studies of predictive logistic regression models. J Clin Epidemiol. 2005;58:475–483.

2. Peduzzi P., Concato J., Kemper E., Holford T.R., Feinstein A. A simulation study on the number of events per variable in logistic regression analysis. J Clin Epidemiol. 1996;49:1373–1379.

3. Ogundimu EO, Altman DG, Collins GS. Adequate sample size for developing predictions models is not simply related to events per variable. J Clin Epidemiol. 2016;76:175–182.

4. Collins GS, Ogundimu EO, Altman DG. Sample size considerations for the external validation of a multivariable prognostic model: a resampling study. Statist. Med. 2016, 35 214–226.

5. Debray TPA, Vergouwe Y, Koffijberg H, Nieboer D, Steyerberg EW, Moons KGM. A new framework to enhance the interpretation of external validation studies of clinical prediction models. J Clin Epidemiol. 2015;68:279–289.
