# Supplementary material for: Predicting short-term interruptions of antiretroviral therapy from summary adherence data: Development and test of a probability model
Source: PLoS One. 2018 Mar 22;13(3):e0194713. doi: 10.1371/journal.pone.0194713 (PMC5864044; doi:10.1371/journal.pone.0194713)
Supplement: S3 Appendix — (DOCX) [file pone.0194713.s003.docx]

**S3 Appendix. Excluded cases and sensitivity analysis**

From the initial cohort of 308 treatment-naïve HIV-infected individuals, we excluded from analysis three categories of participants based on their average adherence. S1 Fig diagrams the case exclusions and the resulting sample size at each decision poin.

We conducted a sensitivity analysis of model performance for the 3 samples: the “comprehensive sample” with no case exclusions, the “intermediate sample” that excludes the completely determined cases – i.e., cases in which an interruption must occur (very high adherence) or cannot occur (very low adherence), and the “narrow sample” that further excludes the cases in the 0.333 to 0.39 average adherence interval. The results are presented in S1 Table.

Prediction performance was strongest in the “comprehensive sample” followed by the “intermediate sample” and then the “narrow sample.” In designing the test of our model, we adopted a conservative approach – *ab initio* choosing the “narrow sample” for our study participants – to avoid tilting the results in favor of the prediction model.

**S1 Table. Sensitivity analysis. Performance metrics of the prediction model with and without the excluded cases.**

| Eq (1) prediction model performance  metrics | **Comprehensive Sample**  N = 308 | **Intermediate Sample**  N = 188 | **Narrow Sample**  N = 185 |
| --- | --- | --- | --- |
| % correctly classified  (95% CI) | 85%  (81% – 89%) | 76%  (69% – 81%) | 73%  (66% – 79%) |
| Sensitivity  (95% CI) | 66%  (56% – 75%) | 67%  (57% – 77%) | 59%  (48% – 69%) |
| Specificity  (95% CI) | 95%  (91% – 97%) | 84%  (75% – 91%) | 87%  (79% – 93%) |
| AUROC  (95% CI) | 0.94  (0.92 – 0.97) | 0.86  (0.81 – 0.91) | 0.85  (0.80 – 0.91) |
| AUROC *k*-fold cross-  validation (*k* = 10)  (95% CI) | 0.93  (0.91 – 0.96) | 0.84  (0.78 – 0.89) | 0.84  (0.78 – 0.90) |
| Brier score | 0.11 | 0.20 | 0.20 |

AUROC: area under the receiver operating characteristic curve
