# Supplementary material for: Predicting short-term interruptions of antiretroviral therapy from summary adherence data: Development and test of a probability model
Source: PLoS One. 2018 Mar 22;13(3):e0194713. doi: 10.1371/journal.pone.0194713 (PMC5864044; doi:10.1371/journal.pone.0194713)
Supplement: S4 Appendix — (DOCX) [file pone.0194713.s004.docx]

**S4 Appendix. Tests for potential confounders**

We fitted two logistic regression models to test for potential confounding factors. In model I, the independent variables were the participant baseline demographic and clinical factors, and the outcome variable was the actual occurrence (yes, no) of an ART interruption of ≥3 days. Model II was the same as model I, but adjusted for our Eq (1) prediction variable. Missing values (alcohol use disorder, n=6; ARV regimen, n=2; CD4 cell count, n=8) were imputed following conventional guidelines [1,2]. The results are presented in S2 Table. Confounding factors were not identified, although patient age was borderline significant.

**S2 Table. Logistic regression. Model I: association of participant demographic and clinical variables with the occurrence of an ART interruption of 3 days or more. Model II: association of participant demographic and clinical variables with the occurrence of an ART interruption of 3 days or more, adjusted for the predicted probability of treatment interruption. N = 185.**

|  | Model I | | Model II | |
| --- | --- | --- | --- | --- |
| Demographic and Clinical Variables | Odds Ratio^a^ | 95% CI | Odds Ratio^a^ | 95% CI |
|  |  |  |  |  |
| Age | 1.04 | 0.99 – 1.10 | 1.08 | 0.99 – 1.17 |
| Gender (reference: female) | 0.81 | 0.29 – 2.26 | 0.79 | 0.21 – 3.04 |
| Education (ref: none) |  |  |  |  |
| primary | 0.27 | 0.02 – 3.18 | 0.41 | 0.02 – 8.85 |
| secondary or above | 0.39 | 0.03 – 5.19 | 0.64 | 0.03 – 15.12 |
| Married (ref: unmarried) | 0.52 | 0.22 – 1.26 | 0.86 | 0.25 – 2.19 |
| Literate (ref: no) | 1.53 | 0.19 – 12.54 | 1.61 | 0.13 – 19.62 |
| Unemployed (ref: no) | 2.41 | 0.88 – 6.58 | 2.78 | 0.77 – 10.14 |
| Distance from clinic in minutes | 1.00 | 0.99 – 1.01 | 1.00 | 0.99 – 1.01 |
| Alcohol use disorder (ref: no) | 0.36 | 0.08 – 1.36 | 0.33 | 0.05 – 1.89 |
| Depression (ref: no) | 1.21 | 0.48 – 3.05 | 0.98 | 0.29 – 3.31 |
| CD4 cell count | 1.00 | 0.99 – 1.00 | 1.00 | 1.00 ­– 1.00 |
| ARV regimen (ref: 3TC/AZT/NVP) |  |  |  |  |
| 3TC/D4T/NVP | 0.38 | 0.12 – 1.23 | 0.35 | 0.07 – 1.64 |
| 3TC/AZT/EFV | 0.79 | 0.22 – 2.81 | 0.59 | 0.16 – 4.69 |
| other ARV regimens | 2.12 | 0.37 – 12.01 | 0.68 | 0.05 – 10.11 |

^a^Dependent variable was the occurrence of a 3-day treatment interruption in the first 90 days of antiretroviral therapy coded so that 0 = no interruptions and 1 = at least one interruption. Missing values (alcohol use disorder, n=6; ARV regimen, n=2; CD4 cell count, n=8) were imputed. 3TC/AZT/NVP: Zidovidine/Lamivudine/Nevirapine; 3TC/D4T/NVP: Stavudine/Lamivudine/Nevirapine; 3TC/AZT/EFV: Zidovudine/Lamivudine/Efavirenz; other ARV regimens: Stavudine/Lamivudine/Efavirenz, Zidovudine/Nevirapine/Tenofovir, Efavirenz/Emtricitabine/Tenofovir, Emtricitabine/Nevirapine/Tenofovir.

1. Rubin DB, Schenker N. Interval estimation from multiply-imputed data: A case study using census agriculture industry codes. Journal of Official Statistics. 1987;3:375-387.

2. Rubin DB, Schenker N. Multiple imputation in health-care data bases: An overview and some applications. Statistics in Medicine. 1991;10(4):585-598.
