# Supplementary material for: Predicting short-term interruptions of antiretroviral therapy from summary adherence data: Development and test of a probability model
Source: PLoS One. 2018 Mar 22;13(3):e0194713. doi: 10.1371/journal.pone.0194713 (PMC5864044; doi:10.1371/journal.pone.0194713)
Supplement: S5 Appendix — (DOCX) [file pone.0194713.s005.docx]

**S5 Appendix. Tests for secular trend**

We tested for a secular trend by comparing the percentage of patients who experienced treatment interruption by the year that ARV therapy began. The results are presented in S3 Table.

**S3 Table. Treatment interruption by participants’ ART start date (year).**

| ART Interruptions  of 3 days or more | 2005  (N = 14)  % | 2006  (N = 57)  % | 2007  (N = 53)  % | 2008  (N = 42)  % | 2009  (N = 17)  % | 2010  (N = 2)  % |
| --- | --- | --- | --- | --- | --- | --- |
| None | 57.1 | 50.1 | 47.2 | 47.6 | 58.8 | 50.0 |
| At least one | 42.9 | 49.1 | 52.8 | 52.4 | 41.2 | 50.0 |

Pearson chi-squared = 1.09, *P* = 0.96, *P* for trend = 0. 89, N = 185.

We also examined the relationship with logistic regression in which the dependent variable was the occurrence of a 3-day treatment interruption. The absence of an interruption was coded as 0 and the occurrence of an interruption was coded as 1. The independent variable was the year ARV therapy began. The OR (odds ratio) was 1.01 (95% CI = 0.78 – 1.30). A secular trend was not detected.
